# Perspectives of clinical stakeholders and patients from four VA liver clinics to tailor practice facilitation for implementing evidence-based alcohol-related care

**DOI:** 10.1186/s13722-023-00429-3

**Published:** 2024-01-10

**Authors:** Elena M. Soyer, Madeline C. Frost, Olivia V. Fletcher, George N. Ioannou, Judith I. Tsui, E. Jennifer Edelman, Bryan J. Weiner, Rachel L. Bachrach, Jessica A. Chen, Emily C. Williams

**Affiliations:** 1 Department of Health Systems and Population Health, University of Washington School of Public Health, 3980 15th Ave NE, Seattle, WA 98195 USA; 2 Health Services Research & Development (HSR&D) Center of Innovation for Veteran-Centered and Value-Driven Care, Veterans Affairs (VA) Puget Sound Health Care System, 1660 South Columbian Way, Seattle, WA 98108 USA; 3 Department of Medicine, University of Washington School of Medicine, 325 9th Ave, Seattle, WA 98104 USA; 4 Yale Schools of Medicine and Public Health, 367 Cedar Street, ES Harkness, Suite 401, New Haven, CT 06510 USA; 5 Department of Global Health, University of Washington School of Public Health, 1959 NE Pacific St, Seattle, WA 98195 USA; 6 Department of Psychiatry, University of Michigan Medical School, 1301 Catherine St., Ann Arbor, MI 48109 USA; 7 Center for Clinical Management Research, VA Ann Arbor Healthcare System, 2215 Fuller Rd, Ann Arbor, MI 48105 USA; 8 Department of Psychiatry and Behavioral Science, University of Washington School of Medicine, 325 9th Avenue, Seattle, WA 98104 USA

**Keywords:** Alcohol, Screening, Alcohol use disorder treatment, Liver, Hepatology, Practice facilitation, Implementation, Qualitative

## Abstract

**Background:**

Unhealthy alcohol use (UAU) is particularly dangerous for people with chronic liver disease. Liver clinics may be an important setting in which to provide effective alcohol-related care by integrating evidence-based strategies, such as brief intervention and medications for alcohol use disorder. We conducted qualitative interviews with clinical stakeholders and patients at liver clinics in four Veterans Health Administration (VA) medical centers to understand barriers and facilitators of integrating alcohol-related care and to support tailoring of a practice facilitation implementation intervention.

**Methods:**

Data collection and analysis were guided by the Consolidated Framework for Implementation Research (CFIR). Interviews were transcribed and qualitatively analyzed using a Rapid Assessment Process (RAP) guided by the CFIR.

**Results:**

We interviewed 46 clinical stakeholders and 41 patient participants and analyzed findings based on the CFIR. Clinical stakeholders described barriers and facilitators that ranged from operations/clinic resource-based (e.g., time and capacity, desire for additional provider types, referral processes) to individual perspective and preference-based (e.g., supportiveness of leadership, individual experiences/beliefs). Patient participants shared barriers and facilitators that ranged from relationship-based (e.g., trusting the provider and feeling judged) to resource and education-based (e.g., connection to a range of treatment options, education about impact of alcohol). Many barriers and facilitators to integrating alcohol-related care in liver clinics were similar to those identified in other clinical settings (e.g., time, resources, role clarity, stigmatizing beliefs). However, some barriers (e.g., fellow-led care and lack of integration of liver clinics with addictions specialists) and facilitators (e.g., presence of quality improvement staff in clinics and integrated pharmacists and behavioral health specialists) were more unique to liver clinics.

**Conclusions:**

These findings support the possibility of integrating alcohol-related care into liver clinics but highlight the importance of tailoring efforts to account for variation in provider beliefs and experiences and clinic resources. The barriers and facilitators identified in these interviews were used to tailor a practice facilitation implementation intervention in each clinic setting.

**Supplementary Information:**

The online version contains supplementary material available at 10.1186/s13722-023-00429-3.

## Background

Alcohol use is a major driver of global mortality and morbidity and is particularly dangerous for people with chronic liver diseases, including both those caused primarily by alcohol (e.g., alcohol-associated liver disease, hepatitis, and cirrhosis) and those for whom concurrent alcohol use is an important cofactor such as hepatitis C virus (HCV) infection, metabolic and alcohol-related liver disease (MetALD), cirrhosis, and hepatocellular carcinoma [1, 2]. For instance, HCV—one of the leading causes of liver disease worldwide [3]—has synergistic interactions with alcohol use resulting in accelerated liver damage and decompensation and higher risk for cirrhosis and hepatocellular carcinoma [4]. In addition, the interaction between metabolic-associated steatotic liver disease (MASLD) and alcohol has recently been officially recognized with the new terminology of MetALD [2].

There are multiple evidence-based practices to address unhealthy alcohol use (UAU) [5]. These include brief intervention—the provision of alcohol-related advice to reduce drinking (commonly referred to as the “harm-reduction” approach [6] and/or to abstain completely, coupled with information regarding the effect of alcohol use on health, often delivered using motivational interviewing techniques—for all patients screening positive for UAU and behavioral therapies and/or medications for those with alcohol use disorders (MAUD) [7–10]. However, ensuring that patients with UAU receive this care in routine clinical settings has been challenging [11–13].

Supporting patients with liver conditions who consume alcohol in reducing or abstaining from alcohol use is an essential component of improving liver health. Liver clinics are an important setting in which to provide effective alcohol-related care. Due to the harm alcohol confers on patients with liver conditions, and the fact that treatment of many liver conditions requires repeated visits, both experts and national organizations, including the American Association for the Study of Liver Disease, have called for the provision of alcohol screening, brief intervention, and behavioral and/or medication treatment as needed for patients with chronic liver disease who drink at unhealthy levels [14, 15]. Prior studies have suggested that evidence-based interventions recommended in primary care and other health settings are appropriate for patients being treated in liver-specific care settings [1, 16–21].

The VA is an important provider of liver care in the U.S., and while it offers an ideal setting for integrating alcohol-related care into liver clinics due to the high prevalence of chronic liver diseases among Veterans who receive care in VA settings [22, 23], liver clinics have not historically been the primary setting in which alcohol-related care is delivered in the VA, and alcohol-related care is not currently required in this setting. However, the VA has a history of strong progress on implementing alcohol-related care into other outpatient settings supported by clinical guidelines outlining the use of behavioral and medication treatments for AUD [9, 24] and a strong foundation in integrating quality improvement efforts into liver clinics. Since the groundbreaking availability of curative treatment for HCV (direct-acting antivirals, or DAAs) in 2014, the VA has treated and cured more than 100,000 Veterans via integrated care improvements led by national clinical leaders and administrators [25].

Though integrating alcohol-related care into liver clinics is a promising strategy to increase access to alcohol-related care among patients who would likely benefit from it, barriers to such care integration remain (e.g., clinical stakeholders in liver clinics may have multiple competing priorities and may thus feel unable to prioritize the changes necessary to integrate alcohol-related care into their practice) [14]. Despite this, research illuminating these barriers from the perspective of key stakeholders (e.g., clinicians, other clinic staff, and patients) is limited. To refine an implementation intervention to facilitate such integration, we conducted semi-structured interviews at four VA liver clinics with clinicians and other clinic staff, as well as patients seen in these liver clinics who reported varying levels of alcohol use at annual screening in any VA setting. The present qualitative study reports findings from interviews to inform the tailoring of a practice facilitation implementation intervention [26].

## Methods

### Overview and guiding framework

Our overall goal was to identify barriers to and facilitators of integrating alcohol-related care into liver clinics to inform the tailoring of a practice facilitation intervention—an evidence-based implementation strategy in which a practice facilitator works with a team to develop strategies to address gaps in care and build clinical capacity to improve health care outcomes [27, 28–30]. We obtained feedback from liver clinic clinicians, other clinic staff, and patients to better understand the unique circumstances associated with providing alcohol-related care in liver clinics. From clinical stakeholders, we specifically sought to learn how each of the four clinics function, understand existing practices for alcohol-related care, and ascertain clinical resources and needs for integrating alcohol-related care in preparation for the implementation intervention. From patients, we sought to understand experiences receiving both alcohol- and liver-related care and their preferences related to receiving alcohol-related care in the liver clinic. Both sets of semi-structured interview guides were developed using the Consolidated Framework for Implementation Research (CFIR). The CFIR was updated in 2022 [31]; as this work was conducted prior to 2022 we used the older version of this framework [32]. The CFIR includes five broad domains: [1] characteristics of intervention (e.g., relative advantage and adaptability; updated in 2022 to “characteristics of the innovation”), [2] outer setting (e.g., patient needs and resources as well as external policies), [3] inner setting (e.g., structural characteristics, culture, and readiness), [4] characteristics of individuals (e.g., preferences, knowledge, and beliefs), and [5] implementation process (e.g., planning, internal implementation leaders, and external change agenda [31, 32].

All procedures were reviewed and approved by the VA Puget Sound Institutional Review Board.

### Setting, study population, and recruitment

The study setting was four urban VA liver clinics located across three states in the Western U.S. These sites were selected based on existing relationships with the research team, expressed willingness to participate, and the number of patients with liver conditions and UAU identified in preliminary research.

#### Clinical stakeholder sample and recruitment

Clinicians and other clinic staff who interact with patients and/or help the clinic to function (e.g., clinic directors, physicians, nurse practitioners, nurses, physician assistants, clinical pharmacists, social workers, fellows or trainees, and front desk staff) were recruited by establishing contact at each site with clinical leaders who then facilitated introductions to other stakeholders. Within clinics, investigators strove to recruit all willing clinical stakeholders [33]. Clinical stakeholders were interviewed during working hours and were not otherwise compensated for their time consistent with VA policies that restrict payment of clinicians for research.

#### Patient sample and recruitment

Data from VA’s Corporate Data Warehouse (CDW)—a relational database that mirrors the electronic health record—were used to identify patients with a documented visit at a participating liver clinic in the prior year who had HCV and/or cirrhosis (see: diagnostic codes indicating eligibility in Additional file 1: Appendix A, Tables A1 and A2) and a documented Alcohol Use Disorders Identification Test Consumption (AUDIT-C) screen [34, 35]. Data were extracted using purposive sampling to increase variation across gender, race, ethnicity, level of alcohol use (based on most recent AUDIT-C score) [36], and liver diagnoses. Patients were excluded who had documented pregnancy, hospice enrollment, cognitive impairment, history of intentional self-harm, and/or no documented telephone number. Eligible participants were recruited via letters and phone calls. At the beginning of each interview, participants were asked for verbal consent. Patient participants were compensated $30 for their time.

### Data collection

Semi-structured interviews were conducted with clinical stakeholders in person or by telephone and all patient interviews were conducted by telephone. All interviews were conducted between April and June of 2019. Interview guide development was guided by the CFIR and designed to elicit feedback on the process of providing alcohol-related care in the liver clinic and on the feasibility of aspects of an initial list of potential strategies for incorporating into a practice facilitation intervention (Additional file 1: Appendix B [37, 38]).

Clinician interview questions covered the characteristics of individuals (e.g., experience, knowledge, and self-efficacy regarding providing alcohol-related care); the characteristics of the intervention (e.g., perceptions of evidence, advantage, and complexity of providing alcohol-related care in the liver clinic setting); the outer and inner settings (e.g., VA policies, perceptions of readiness of the clinic to integrate alcohol-related care); and the process of implementation (i.e., elements of the practice facilitation intervention such as clinic champions, design meetings, and educational handouts for patients). Front desk staff were not asked questions pertaining to direct clinical care. Patient interview questions included closed-ended questions regarding sociodemographic and clinical characteristics and then covered the categories of patient experience and knowledge of alcohol-related care, patient treatment preferences, and desired support from the clinic. All interviews were digitally recorded and transcribed verbatim.

### Data analysis

Patient participant sociodemographic and clinical characteristics were summarized. Post-transcription, all interviews were analyzed using the Rapid Assessment Process (RAP), an intensive approach using triangulation and iterative analysis to quickly develop understanding and support intervention development [39–41]. Per RAP methods, each interview was distilled into a 1–2-page template summarizing responses to each question, including representative quotations, based on the CFIR framework. The collaborative nature of RAP makes it more efficient than traditional qualitative methods, while remaining comparable to traditional methods with ~ 80% overlap in findings [42]. RAP templates were reviewed against transcripts for accuracy by a second team member and presented to the full investigative team for feedback. Individual templates, along with summaries of key themes and feedback from the project team, were used to plan facilitation strategies. Final themes and practice facilitation strategies were reached by consensus with the full investigative team and are presented here with prototypical examples.

## Results

### Sample characteristics

Forty-six clinical stakeholders were interviewed across sites. (Ns: 14, 12, 8, and 13). The response rates at the clinics were 88%, 92%, 100% and 100%.

The characteristics of the 41 (site-specific Ns: 12, 10, 11, and 8) patient participants are summarized in Additional file 1: Appendix A, Table A3. Demographic data in this study was self-reported by patient participants during interviews. The sample mostly self-reported as male (85.4%) and majority White (58.5%), though it also included many patients self-reporting as Black (12.2%), American Indian (7.3%), and multi-race (7.3%). Most had some college/technical school or more (70.7%). Close to half were divorced (43.9%) and 19.5% were working either full- or part-time. The mean AUDIT-C score was 5.0 (SD = 4.0), consistent with unhealthy alcohol use [35, 43, 44]. Of the 14 participants with current non-drinking (AUDIT-C = 0), all described some prior alcohol use, and 10 described having UAU and/or reported experiencing problems related to alcohol in the past. Qualitative themes are summarized and mapped to the CFIR domains in Additional file 1: Appendix A, Table A4.

### Outer setting

One facilitator was identified within the Outer Setting domain, reported by clinical stakeholders, not patients.

#### Facilitator (clinical stakeholder): VA policy

The VA’s policy to not withhold HCV treatment for patients with active alcohol use was described as a facilitator to providing alcohol-related care in liver clinics because it increases the likelihood that patients with alcohol use-related issues are present in the liver clinic. One participant commented:

“*[The VA policy that allows treatment of HCV in people with active substance use] has been really helpful because we’ve had a number of people that have successfully been cured, or cleared of [HCV], and they’re not 100% abstinent or sober from substances. So we’ve had people that continued to consume alcohol that have been cleared. We’ve had individuals who’ve used [methamphetamine] that have been cleared. It’s just really encouraging to see active substance use isn’t a barrier to getting people on medication.*”

### Inner setting

Five barriers and four facilitators were reported by clinical stakeholders in the Inner Setting domain. Two barriers and one facilitator were reported by patient participants.

#### Barrier (clinical stakeholder): provider resources

Clinical stakeholders worried that time and space were inadequate for incorporating alcohol-related care. One said:

“*Obviously the drawback is time, right? This isn’t something I feel should be rushed. If you’re going to have this conversation with a patient, it shouldn’t be a rushed conversation. So just making sure that patients are scheduled accordingly to allow for the discussions to occur, I think is important, and I think is a potential barrier. Currently, because patients are scheduled in shorter time blocks. You may not have adequate time to discuss and answer questions and talk about it in the detail you would like to.*”

They also thought additional appointment time may be required to review educational handouts included in the intervention, which may be incompatible with the shorter time blocks described by providers. One shared, “*I would say probably, I don’t know, the top 10% of VA patients, I feel like I could hand them our patient pamphlet about cirrhosis or HCV and they’re going to understand it, but otherwise, I find that I have to kind of talk them through it*.”

Clinical stakeholders also expressed concern about inadequate staff resources in the clinics, specifically in relation to additional staffing such as mental health specialists to support the integration of alcohol-related care. One participant shared:

*“It’s really, really hard. Because right now we don’t really have access to somebody for substance abuse [sic]. I’ll be honest, I can think of the last couple of patients, before when there was someone in place who could be seen right away, who I could coordinate their appointments with my appointments, it was very easy. I’d say for the last three years or so, we’ve lost that, so now I just have to go the regular route of a mental health consult.*”

Another participant shared that that there is also a lack of the necessary nursing staff to provide injectable naltrexone (vivitrol) and other alcohol-related services: “*We don’t do Vivitrol in [the] hepatology clinic because we don’t have a nurse that can do the injections.*”

#### Barrier (clinical stakeholder): clinic conditions and existing Workflows

Clinical stakeholders also lacked a standard approach to screening for unhealthy alcohol use and following up on positive screens. One participant noted, “*The first thing that we try to do is assess alcohol use. I have to admit I’m guilty of not doing it in a systematic way, I don’t routinely use an instrument… but I do routinely ask about alcohol use*.”

Providers described that their approach often involved passing along the alcohol-related care to someone else: “*In the liver clinic we just recognize it [unhealthy alcohol use], identify it, [and] do sort of leave it to another provider*.”

Clinical stakeholders also described the lack of connection to other resources, such as VA specialty addictions care and a clear referral process, as a barrier. One participant noted:

“*I know there are resources on campus here, there’s the [domiciliary] and some inpatient facilities, and to be honest, I don’t exactly know how to always get my patient there, so sometimes that’s a little bit of a hurdle; we know we have resources, but … there’s a communication gap between what they do and how we can access those resources.*”

At teaching clinics with trainees, an additional barrier emerged: trainee turnover, especially as it relates to training and implementation. One participant described:

“*I would say I would not [prescribe AUD medications] at this liver clinic… because it’s so fellow-run, and so resident-run, that there’s very little continuity… if you ask any liver patient here who their liver doctor is, they would have no idea, unless they’re a transplant patient. And the reason why is because there’s so much turnover in regard to residents and fellows every single month.*”

In this context, hesitance to treat AUD with medication was tied to the clinic’s staffing structure and the rotational nature of fellow- and resident-led care.

#### Facilitator (clinical stakeholder): clinic priorities

In general, clinical stakeholders described three ways in which integrating alcohol-related care to the liver clinic aligned with clinic priorities as a facilitator. First, leadership support facilitated the possibility of integrating alcohol-related care; clinical stakeholders at liver clinics believed that their leadership teams would be supportive of promoting alcohol-related care. One participant described, “*They’re willing to try new things. And I think having [clinic director] at the helm, he’s super Innovative, he’s always, he’s so progressive in his way of thinking about how we’re treating veterans.*”

Second, clinical stakeholders viewed alcohol interventions as a natural fit for liver clinic care because they are aligned with the clinic’s mission and available resources. One participant said:

“*Many [liver clinic patients] are there because of their alcohol use, and it just seems like it would be natural for alcohol use disorder to be addressed in the liver clinic as part of that comprehensive liver care, but I feel like most hepatologists and GI providers really don’t view alcohol use disorder as something in their treatment realm… so I think you could change that mindset and show that this is totally feasible without too many extra resources.*”

Third, clinician and staff participants recognized the success of VA’s prior quality improvement initiatives as a facilitator. One participant reported, “*Because we’ve done such a great job with [HCV], we’re now opening up our clinic, so there is the ability to do walk-in perhaps… We have the space and the ability right now in our clinic in order to make any changes that could be done*.” Previous clinic experience with the HCV quality improvement initiative paves the way for quality improvement to remain a part of clinic culture and norms.

#### Facilitator (clinical stakeholder): availability of interdisciplinary team

Clinical stakeholders recognized the potential for capitalizing on the availability of an interdisciplinary team in the clinic, including varied staff such as pharmacists and social workers, to facilitate successful integration of alcohol-related care. One participant noted:

“*Maybe the medications will help, but the social issues that surround may be the reason that they’re drinking so much. That’s where I depend upon the rest of the team; the social worker helps, the psychologist helps. They can address some of those other issues. As a pharmacist I’m a little different in that sense. But that’s where you depend on the team. I think alcoholism is multifactorial, you can’t just expect medications to be the sole solution to the whole thing.*”

Another provider shared:

“*We used to have a pharmacist in the clinic, of course in conjunction with [psychiatry], to help us with that patient population. I think social workers are important, in addition to the clinical aspect there’s a social aspect they can help with as well. We need pharmacy’s support, to give us a pharmacist. And we need to have a [psychiatry] person to supervise, with pharmacy service. I think it would be so helpful to have a social worker in the clinic as well.*”

#### Barrier (patient): patient experience of receiving care

Though patients generally described positive experiences with the liver clinic, their reviews of overall VA services were more mixed, especially regarding long wait times. As patient trust is an important facilitator of patient-centered alcohol care, mixed sentiments towards overall VA care may serve as a barrier to patients seeking alcohol-related care.

Some patients also described a lack of continuity of care and how it sometimes challenged their provider’s ability to attend to their concerns. One patient noted:

“*It just seems like you get a new doctor all of the time. And nothing is worse than going into an appointment, and having the doctor ask you, ‘what brings you in today?’…you should know, you’ve got my file in front of you…If you ask that question, then it leads me to believe that, you know, you don’t care, you’re lazy, or you don’t know what you’re doing.*”

This concern was especially pertinent in teaching clinics as opposed to non-teaching clinics due to the rotating nature of staffing.

#### Facilitator (patient): positive patient experience at liver clinic

Patients reported positive experiences receiving care at liver clinics which could facilitate their receiving alcohol-related care during these visits. One said, “*The people are knowledgeable, they seem to care about the patient and what they’re doing…I felt they weren’t just doing a job, I felt like they were concerned and wanted to accomplish something*.” Another said, “*when I’ve been to the liver clinic, they’ve all been very nice, very professional, forthcoming. They try to talk you through things, and if you don’t understand something they’ll make it so that you can understand, you know? They made it easy for me*.”

### Individual characteristics

Two barriers and one facilitator were reported by clinical stakeholders in the Individual Characteristics domain. One barrier and two facilitators were reported by patient participants.

#### Barrier (clinical stakeholder): provider beliefs associated with provision of alcohol-related care in the liver clinic

Some clinical stakeholders reported the concern that treatment of liver conditions may be futile among patients that drink, and that it is not the role of the liver clinic to address unhealthy alcohol use. Regarding the concern of futility, some providers expressed a preference that treatment be reserved for patients who are abstinent. While this view was not universal across providers, it arose multiple times. One participant said: “*I don’t want to sound like I don’t want to deal with them, but it really is a futile thing to try to take care of somebody as they’re drinking their liver to death, and they can’t or won’t stop. It’s just, I mean, there’s really nothing you can do*.”

Regarding the liver clinic’s role in treating UAU, one provider noted lack of time preventing clinicians from engaging with alcohol-related care: *“Hepatologists are busy and don’t have time or feel it isn’t their responsibility to help patients with unhealthy alcohol use.”*

#### Barrier (clinical stakeholder): lack of training related to alcohol-related care

Throughout interviews, clinical stakeholders reported needing additional training to be comfortable implementing alcohol-related care in the liver clinic. some providers especially felt concern toward medication. One participant shared:

“*I’m not anti [medication], I think that if a person who is well-trained, who knows how to manage these medications and the pitfalls thereof, or the advantages obviously, thereof, I would totally be open to it. I was just not necessarily trained to use those medications, so I don’t know that I would be the most appropriate person to do that… [I would need] an understanding of what monitoring I would need to do, how much follow-up they would need, maybe just sort of predictors of success.*”

#### Facilitator (clinical stakeholder): provider interest and enthusiasm for providing alcohol-related care

Many providers strongly believed in the importance of addressing alcohol use and confronting the stigma of alcohol use. One participant said:

“*I think of substance use like a disease, like any other. We would never say to a patient with heart disease, ‘sorry, you’re not going to be allowed to get Plavix because you eat too much salt’, or, ‘you can’t get treatment for your diabetes until you lose weight’. There’s all of these other behavioral aspects to medicine where we aren’t imposing an arbitrary requirement that patients behave in a certain way in order to deserve clinical treatment. I feel that substance use is a very value-laden kind of condition, so there’s a tendency to blame patients for having addictions, and punishing them in ways that hurt their overall health. I’ve never believed in restricting [liver] treatment for people who use injection drugs, or alcohol use.*”

Additionally, clinical stakeholders reported interest and experience with alcohol use-related treatment options. The same provider shared:

*“Besides referrals to other places, I come from a Primary Care background where I am completely comfortable prescribing Gabapentin, or Baclofen. Or Naltrexone, orally…So I can refer patients or I can get them started on some kind of pharmacotherapy. I think that some of the other providers are less comfy with that because they don’t have that training background, and the comfort level that we have in Primary Care with these drugs. But I’m, I give that stuff out like crazy. I feel there’s very low harm and it can potentially help them with their alcohol cessation.”*

#### Barrier (patient): negative judgment

Patients noted experiencing negative judgement from providers. Some patients shared that they felt judged by liver providers regarding actual or perceived alcohol use and believed providers would not help them unless they abstained. One patient shared, “*I keep telling him that I don’t drink, I don’t know if they hear it or if they just think that I’m a recovering alcoholic… I guess maybe a lot of people are assuming that I have Hepatitis C, I must have got it from drugs or alcohol.*” Another noted, “*It would be great if somebody steered you in the right place where to go, instead of, you know, passing judgement or making weird faces or something*.”

#### Facilitator (patient): comfort talking about alcohol use with providers

Patients reported trust and comfort discussing alcohol use with providers. When asked whether liver clinic providers have a role in alcohol-related care, one patient said: “*I think definitively there’s a role, because that’s really how I was introduced to recovery*.*”* Another, describing a conversation with their provider, responded:

“*It went well. He asked me about the education part of it, I didn’t know that having liver issues could lead to some of the other ailments, like cancer and all of that other stuff. They really helped me out with the education portion, to let me know that it was wiser to give up on alcohol. I didn’t think it was that damaging to the body, especially with the amount that I was using. Even prostate, and high blood pressure, both of those are really bad. Yeah, alcohol is bad… they helped me out a lot. I had no idea that it could cause as much problems as it does.*”

#### Facilitator (patient): openness to alcohol interventions

Patients expressed openness to alcohol use interventions, so long as they are educated by providers on treatment options. One patient noted not feeling as though they knew the full extent of treatment options available to them: “*If I knew more about the medications that I could take, I probably would’ve tried them out. But I haven’t directly, I haven’t been offered any medications exactly for drinking.*”

### Intervention characteristics

One barrier and two facilitators, all reported by patients, were identified in the Intervention Characteristics domain.

#### Barrier (patient): perception that providers do not understand barriers faced by patients

Patients reported feeling not understood by providers and specifically, they reported perceiving that providers do not understand the barriers they face to changing drinking and accessing treatment, with one saying:

“*It was easy for him to say, ‘this is what you need to do, and you need to go to this place to have it done’, and it sounded all simple. But for me, it was very daunting. Very hard for me. It would be easier for me to stay home and drink than to get on the bus, go across the river, which would take me hours, and go deal with people that I wasn’t entirely sure that I trusted or entirely understood what I was going through.*”

#### Facilitator (patient): variation in alcohol-related treatment options

Interviews revealed that patients had a variety of opinions and perspectives regarding alcohol treatment and that variation in alcohol-related treatment choices and more choice regarding drinking (e.g., abstinence versus cutting back) can facilitate willingness to reducing drinking. For example, one patient shared positive experiences attending Alcoholics Anonymous (AA). They said, “*AA is basically peer therapy. I think it’s extraordinarily helpful, especially early in recovery… it doesn’t demand much from you as an individual, you can just attend*.*”* Another patient, however, shared that AA did not resonate with them. They noted, “*With AA, I think it’s too extreme. I think I don’t need to go out every single time and tell people I’m an alcoholic. That’s not the focus. That’s not my intent for being at this particular gathering, right? Alcohol doesn’t define who I am.”* Provider acknowledgement that not all patients benefit from the same additional services may help to facilitate more patient-centered treatment plans.

Regarding treatment goals, one patient described having success in reducing drinking after receiving harm reduction-based guidance from their providers: “*The first one said that I should cut back, and I said, ‘you mean stop?, and she goes ‘do the best that you can’. And the second one told me to continue what I was doing… I hadn’t completely stopped but I cut back a lot.*”

#### Facilitator (patient): clear, comprehensive advice

Patients also expressed interest in receiving comprehensive and clear advice regarding alcohol use. Patients shared that they wish they had a better understanding of the impact drinking had on their livers and that such help could support them in engaging in alcohol related care. One shared about the type of information they would like to receive from their provider:

“*And letting you know more about what it’s doing to your body. That, they don’t really emphasize much on that, most of them just kind of skim over it a little bit, they don’t really emphasize what it’s really majorly doing to your body. And most of us, we really do not have an idea totally of what that stuff did to us at times, even though we continue doing it, because that’s just how we are.*”

Another patient noted, “*Probably if they had informed me of the full effect of a damaged liver, what I would go through or possibly might go through, probably would go through. If maybe I had seen another live person at the stage that I was in or am in. That may have made a difference.*”

### Implementation process

One barrier and three facilitators were reported by clinical stakeholders in the Implementation Process domain.

#### Barrier (clinical stakeholder): lack of time for practice facilitation

Clinical stakeholders repeatedly cited lack of time to commit to the practice facilitation process (e.g., attending meetings with the practice facilitator) as a barrier to engaging with the intervention.

#### Facilitator (clinical stakeholder): key roles

Clinical stakeholders expressed enthusiasm for multiple components of the proposed practice facilitation intervention. For one, multiple interviewees were enthusiastic about the use of a practice facilitator and the identification of clinic champions. One participant shared, “*I think that model, they’ve already kind of assimilated to that, having them being trained and having local champions. They have had capacity to do more of these local champion types of workgroups, and that’s something that I think is encouraged*.” Another said, “*I think the idea of establishing someone in the clinic to bang the drum at the local level is a good idea*.”

#### Facilitator (clinical stakeholder): convenient meetings

Clinical stakeholders reported that they thought that utilizing existing meeting times, keeping meetings short, and utilizing remote-meeting technology could help facilitate implementation. One provider described it as, “*Everyone is so busy. If they’re monthly, and they’re short meetings you’ll get more buy in*.”

#### Facilitator (clinical stakeholder): continuation of already-used practices

Specifically, in response to our asking about the usefulness of a patient educational handout, clinical stakeholders generally felt they would aid them in engaging with patients regarding alcohol use, and some clinics reported already using or having previously used educational handouts. One participant noted, “*I remember there was a time when a handout came along and the fellows were very excited, it was helpful for them to be able to engage in conversations because of the handout*.”

## Discussion

In this qualitative study with clinical and patient stakeholder participants from four VA liver clinics, we identified both barriers and facilitators to integrating evidence-based alcohol-related care into liver clinics. Barriers and facilitators identified were observed across all of the CFIR domains and ranged from operations, clinic resource, and clinic policy-based to individual perspective and preference-based.

Many barriers and facilitators identified in liver clinics were similar to those that have been identified in other settings, namely primary care (e.g., time, resources, role clarity, stigmatizing beliefs) [33, 45–47]. However, some barriers—fellow-led care and lack of integration of liver clinics with addictions specialists—and some facilitators—the presence of quality improvement staff in clinics and other integrated pharmacists and behavioral health specialists—emerged uniquely in liver clinic settings. The strong opinions providers expressed regarding liver care and alcohol use may serve as either barriers or facilitators and may also be specific to liver clinic settings.

These findings informed our tailoring of a practice facilitation implementation intervention to integrate alcohol-related care into liver clinics. Patient reports that that they felt comfortable with their liver providers suggested that the intervention should build upon established patient-provider relationships to facilitate discussions of alcohol use and treatment. On the flip side, hearing from some patients that they felt judgement from their providers reinforced the idea that interventions should be patient-centered; practice facilitation should include stigma-reduction efforts and trainings on patient-centered scripts for providers. Patients also expressed interest in receiving clear feedback from their providers about the health consequences of alcohol use, different types of resources for receiving treatment, and direct advice regarding alcohol use reduction/abstinence. Similarly, clinical stakeholders expressed interest in learning more about alcohol treatment and resources. Together these findings suggested that the intervention should include encouragement of the provision of information and educational materials regarding the variety of resources and options available for treating alcohol use and information about shared decision making, an approach to treatment that is widely recommended for alcohol-related care [48]. Finally, we found that lack of time was a barrier not only to providers offering alcohol-related care but also to practice facilitation. Clinic teams suggested using existing clinic meeting times for the practice facilitation intervention in order to avoid losing time to perform other clinic duties or to provide care. It remains unclear whether having separate, dedicated time for practice facilitation may result in better outcomes and more sustained change compared to using existing meeting time for practice facilitation, and future research could compare these two approaches.

Both across and within clinics, there was variation in reported provider feelings towards treating patients with active alcohol use. Some providers expressed strong sentiments that it is important to offer liver care to all patients regardless of current alcohol use status, while others felt that treating patients for alcohol-related liver conditions while they are still actively using alcohol is likely a “futile” effort that would render liver treatment “moot”. The latter of these perspectives reflects substance use stigma—a common theme in efforts to further integrate evidence-based care for alcohol and other substances into medical settings [33, 45, 49–54]—which should be addressed via implementation strategies such as further education and normalizing the provision of alcohol-related care [37, 39, 54]. In sum, these discrepant findings highlight the importance of tailoring implementation interventions—and practice facilitation in particular—to either capitalize on or address local clinical culture and individual-level beliefs.

Though many of the findings were consistent across the four liver clinics, some were clinic-specific and occasionally reflected opposing viewpoints on the same topic. For instance, leadership at the clinic with many fellows and trainees had a unique set of concerns due to the nature of medication-based alcohol treatment and the regular follow-up required, which they were not sure would be possible with their staffing model. Continuity of care was also an issue that arose in patient interviews, with patients noting that not having a consistent provider across visits presented challenges in attending to their concerns. Again, these findings highlight the importance of tailoring implementation efforts to each clinic’s clinical culture and workflow, especially when supporting multiple clinics in integrating evidence-based care. Further research is needed to understand whether practice facilitation can, indeed, capitalize on facilitators and address barriers identified in the present study to successfully implement evidence-based alcohol-related care into liver clinics [14].

Limitations of this study should be noted. First, this is a qualitative study of four liver clinics within the national VA healthcare system. Findings resulting from qualitative inquiry are not generalizable, though they do provide insights that could inform future quantitative research. Findings may also have limited generalizability outside of these four specific clinics and/or outside of VA liver specialty settings. Additionally, given that participating liver clinics were connected with earlier VA quality improvement efforts to increase HCV treatment among Veterans, findings might not be generalizable to liver clinics without prior quality improvement experience.

## Conclusions

The present study identified unique barriers and facilitators to integrating evidence-based alcohol-related care into VA liver clinics from the perspective of both clinical stakeholders and patients in order to inform the tailoring of a practice facilitation intervention—an evidence-based strategy for implementation that is relatively novel to specialty settings. Given the synergistic interactions between alcohol use and liver conditions—including alcohol-attributable conditions or conditions that are alcohol-associated—liver clinics are an important setting in which to provide alcohol-related care. Future research is needed to evaluate whether the resulting intervention effectively supported the integration of alcohol-related care into liver clinics in ways that influenced both care and clinical outcomes.

### Supplementary Information

**Additional file 1.** **Appendix A.** Patient inclusion criteria, diagnostic codes, and results. **Appendix B.** Interview guides.

## Data Availability

Data are not publicly available due to institutional rules regarding data sharing. Consistent with VA policy, the data cannot be made available.

## Abbreviations

UAU: Unhealthy alcohol use
VA: Veterans Health Administration
CFIR: Consolidated Framework for Implementation Research
RAP: Rapid Assessment Process
HCV: Hepatitis C virus
AUD: Alcohol use disorder
CDW: Corporate Data Warehouse
AUDIT-C: Alcohol Use Disorders Identification Test Consumption
MAUD: Medication for alcohol use disorder
