# Supplementary material for: Perspectives of clinical stakeholders and patients from four VA liver clinics to tailor practice facilitation for implementing evidence-based alcohol-related care
Source: Addict Sci Clin Pract. 2024 Jan 10;19:3. doi: 10.1186/s13722-023-00429-3 (PMC10782537; doi:10.1186/s13722-023-00429-3)
Supplement: Supplementary file 1 — Additional file 1. Appendix A. Patient inclusion criteria, diagnostic codes, and results. Appendix B. Interview guides. [file 13722_2023_429_MOESM1_ESM.docx]

**Appendix A – Patient inclusion criteria, diagnostic codes, and results**

| **Table A.1. Inclusion criteria** |
| --- |
| - Age 18+ - Had a documented visit to the clinic in the past year - Had a documented AUDIT-C screening score - Had past year AUD diagnosis or AUDIT-C score indicating unhealthy alcohol use (note: could have a lower AUDIT-C score if they had AUD diagnosis) - Past year diagnosis of HCV and/or cirrhosis |

**Table A.2. Diagnosis Codes Used to Identify Patient Sample**

| ***AUD diagnosis codes*** | | |
| --- | --- | --- |
| - F10.10 - F10.120 - F10.121 - F10.129 - F10.14 - F10.150 - F10.151 - F10.159 - F10.180 - F10.181 - F10.182 | - F10.188 - F10.19 - F10.20 - F10.220 - F10.221 - F10.229 - F10.230 - F10.231 - F10.232 - F10.239 - F10.24 | - F10.250 - F10.251 - F10.259 - F10.26 - F10.27 - F10.280 - F10.281 - F10.282 - F10.288 - F10.29 |
| ***HCV diagnosis codes*** | | |
| - B17.10 - B17.11 - B18.2 - B19.20 - B19.21 - Z22.52 | | |
| ***Cirrhosis diagnosis codes*** | | |
| - K70.30 - K74.69 - K74.60 - K74.3 - K74.4 - K74.5 - K71.7 - E83.110 | | |

| **Table A.3. Demographics of Patient Stakeholders Recruited after Receiving Care in VA Liver Clinics** | |
| --- | --- |
|  | **N (%)** |
| **Clinic sites** |  |
| Puget Sound | 12 (29.3) |
| Portland | 10 (24.4) |
| Long Beach | 11 (26.8) |
| Greater Los Angeles (GLA) | 8 (19.5) |
| **Gender** |  |
| Male | 35 (85.4) |
| Female | 6 (14.6) |
| Other | 0 (0) |
| **Race/Ethnicity** |  |
| White | 24 (58.5) |
| Black | 5 (12.2) |
| Hispanic/Latino | 1 (2.4) |
| AI/AN | 3 (7.3) |
| Asian | 1 (2.4) |
| Multiple Races | 3 (7.3) |
| Unknown | 4 (9.8) |
| **AUDIT-C** |  |
| 0 | 14 (34.1) |
| 1-3 | 7 (17.1) |
| 4-6 | 6 914.6) |
| 7-12 | 14 (34.1) |
| **Liver condition** |  |
| Hepatitis C (HCV) | 10 (24.4) |
| Cirrhosis | 18 (43.9) |
| HCV + Cirrhosis | 13 (31.7) |
| **Marital status** |  |
| Never married | 7 (17.1) |
| Married | 11 (26.8) |
| Divorced | 18 (43.9) |
| Separated | 3 (7.3) |
| Widow | 2 (4.9) |
| **Employment** |  |
| Full-time | 5 (12.2) |
| Part-time | 3 (7.3) |
| Unemployed | 5 (12.2) |
| Disabled | 11 (26.8) |
| Retired | 17 (41.5) |
| **Education** |  |
| Some high school | 0 (0) |
| High school/GED (Graduate Equivalent) | 12 (28.3) |
| Some college | 19 (46.3) |
| College | 5 (12.2) |
| Postgraduate training or degree | 5 (12.2) |

**Table A.4. Barriers and Facilitators Identified from Clinical Stakeholders and Patients from Four VA Liver Clinics in the West United States, Organized by the Broad Domains of the Consolidated Framework for Implementation Research (CFIR)**

|  | **CLINICAL STAKEHOLDERS** | | **PATIENTS** | |
| --- | --- | --- | --- | --- |
| **CFIR Domain** | **Facilitator** | **Barrier** | **Facilitator** | **Barrier** |
| **Outer Setting** | **VA policy:** the VA’s policy not to withhold HCV treatment for patients with active alcohol use |  |  |  |
| **Inner Setting** | **Clinic priorities:** Leadership support | **Provider resources:** Inadequate time and space | **Patient experience at liver clinic:** Positive experience receiving care at liver clinic | **Patient experience receiving care:** mixed reviews of overall VA services |
|  | **Clinic priorities:** Alcohol interventions aligned with existing resources and goals | **Provider resources:** Inadequate staff resources |  | **Patent experience receiving care:**Lack of continuity of care |
|  | **Clinic priorities:** Success of prior quality improvement initiatives | **Clinic conditions and existing workflows:** Lack of standard approach to screening for unhealthy alcohol use and follow up |  |  |
|  | **Interdisciplinary team:** Availability of interdisciplinary team | **Clinic conditions and existing workflows:** Lack of coordination to other resources |  |  |
|  |  | **Clinic conditions and existing workflows:** Trainee turnover |  |  |
| **Characteristics of Individuals** | **Provider interest and enthusiasm for alcohol-related care:** Belief in the importance of addressing alcohol use and confronting stigma of alcohol use | **Provider beliefs associated with provision of alcohol-related care in liver clinic:** Concern that treatment of liver conditions may be futile among patients that drink | **Comfort talking about alcohol use with providers:** Trust and comfort discussing alcohol use with providers | **Negative judgement:** Negative judgement from providers |
|  | **Provider interest and enthusiasm for alcohol-related care:** Interest and experience with alcohol use-related treatment options | **Provider beliefs associated with provision of alcohol-related care in liver clinic:** Belief that it is not the role of the liver clinic to address unhealthy alcohol use | **Openness to alcohol use interventions**: Openness to alcohol use interventions, so long as they are educated by providers about treatment options |  |
|  |  | **Lack of training related to alcohol-related care:** Need for additional training |  |  |
| **Characteristics of Intervention** |  |  | **Variation in alcohol-related treatment options:** Variation in alcohol-related treatment choices can facilitate willingness to reduce drinking | **Perception that providers do not understand barriers:** Perception that providers do not understand the barriers they face to changing drinking and accessing treatment |
|  |  |  | **Clear, comprehensive advice:** Interest in receiving comprehensive and clear advice regarding alcohol use |  |
| **Process of Implementation** | **Key roles:** Use of practice facilitator and identification of clinic champions | **Lack of time for practice facilitation:** Lack of time for practice facilitation meetings |  |  |
|  | **Convenient meetings:** Utilizing existing meeting times and remote-meeting technology |  |  |  |
|  | **Continuation of already-used practices:** Clinics already using educational handouts |  |  |  |

**Appendix B – Interview guides**

**Supplemental Material B.1. Patient Interview Guide**

| *Section A. Demographics*  ***Interviewer:*** *First, I will ask you some questions about your background.*   1. **What is your gender?**     1. Male (1)    2. Female (2) 2. **What is your current marital status?**     1. Never Married (1)    2. Married/Living as married (including cohabitating and partnered (2)    3. Separated (3)    4. Divorced (4)    5. Widow(er) (5) 3. **Are you Hispanic or Latino?**    1. No (1)    2. Yes (2) 4. **What race do you consider yourself?**     1. American Indian or Alaskan Native (1)    2. Asian (2)    3. Native Hawaiian or Other Pacific Islander (3)    4. Black or African American (4)    5. White (5)    6. Bi-racial or multi-racial (6) 5. **What is your current employment status?**     1. Homemaker (give usual occupation of spouse: ________) (1)    2. Disabled (give usual occupation: __________) (2)    3. Unemployed (3)    4. Retired (4)    5. Currently working full-time (give usual occupation: ________) (5)    6. Currently working part-time (give usual occupation and average hours per week_________) (6)    7. Student (7)    8. Other (specify: __________) (8) 6. **What is the highest grade or degree you have competed in school?**     1. 8^th^ grade or less (0-8) (1)    2. Some high school (9-11) (2)    3. High school graduate or GED (12) (3)    4. Some college or technical school (13-15) (4)    5. College graduate (16) (5)    6. Postgraduate Training or Degree (17+) (6)   *Section B. Alcohol consumption*  ***Interviewer:*** *Now, I am going to ask you about your drinking in the past year.*   1. **How often have you had a drink containing alcohol in the past year?**     1. Never *(0 points)*    2. Monthly or less *(1 point)*    3. 2 to 4 times per month *(2 points)*    4. 2 to 3 times per week *(3 points)*    5. 4 or more times a week *(4 points)*   *[If answer “never” skip the next two questions]*   1. **How many drinks containing alcohol did you have on a typical day when you were drinking in the past year?**    1. 1 or 2 *(0 points)*    2. 3 or 4 *(1 point)*    3. 5 or 6 *(2 points)*    4. 7 to 9 *(3 points)*    5. 10 or more *(4 points)* 2. **How often did you have 6 or more drinks on one occasion in the past year?**     1. Never *(0 points)*    2. Less than monthly *(1 point)*    3. Monthly (*2 points*)    4. Weekly *(3 points)*    5. Daily or almost *(4 points)*   *Enter total score*: _____  *Part C. Open-ended questions*  ***Interviewer:*** Now, I am going to ask about your health, and your experience at VA and [*clinic name*].   1. **What has your experience in the Hepatology/liver clinic been like?**    1. In what ways are you getting what you need from VA? In what ways are you not? 2. **When you are receiving healthcare, what do you feel you need to make important treatment choices?**      1. **Tell me about your experiences with drinking alcohol. What, if any, impact has it had on your life? On your health?**    1. What about other substance use? 2. **Have you ever sought help for your drinking or other substance use?**    1. [*If yes*] Where did you look for help?    2. [*If yes*] What type of help felt available to you?    3. [*If yes*] What helped? What didn’t help? 3. **If you haven’t sought help but have considered it, what information might you need or want to make a decision about seeking help?**    1. Who might you want to help you consider your options (e.g., clinicians, family, friends, peers)? 4. **Have you and your doctor ever talked about your drinking? What can you remember about those conversations?**     1. How did the conversation go? Was it easy? Was it hard? 5. Have you and your liver doctor ever talked about your drinking? What can you remember about those conversations?    1. How did the conversation go? Was it easy? Was it hard? 6. **What role could a health system have in making it easier for you and others to get help with your drinking when you had concerns about your drinking? By health system we mean your doctor, nurse or any health professional or medical staff.** 7. **There are several effective treatments & approaches that help people change their drinking. They include counseling interventions, medications, and 12-step or other self-help groups. None has been shown to consistently work better than any other.**     1. If you had concerns about your drinking, what would help you seriously consider one of these options?    2. What would you like to know about any of these options?    3. Would knowing about these options have changed anything you’ve done to seek help for your drinking? 8. **If you also use another substance, what kind of support would you like from your healthcare providers?** 9. **What else would you like to tell us?** |
| --- |

**Supplemental Material B.2. Clinical Stakeholder Interview Guide**

| ***Interviewer:*** *I am now going to ask you 4 short questions about your clinical training and position.*   1. **What is your clinical training?**    1. Medical Doctor (MD)    2. Doctor of Osteopathy (DO)    3. Nurse Practitioner (NP)    4. Advanced Practice Registered Nurse (ARNP)    5. Registered Nurse (RN)    6. Physician Assistant (PA)    7. Other: _____________ 2. **What is your specialty?** 3. **How many years have you worked in this clinic?** 4. **About how much time do you spend working directly with patients each week?**   ***Interviewer:*** *Now I’m going to ask you a few questions about your role in the clinic and your experience with and thoughts about treating patients with liver conditions and substance use.*   1. **Please describe your general role in the clinic.** 2. **What do you think about VA’s stance or policies related to treating Hepatitis C or other liver diseases among patients with substance use?** 3. **How do you work with patients with unhealthy alcohol use or alcohol use disorder in your practice? What is your approach?**    1. What level of alcohol use do you consider concerning for your patients?    2. How can you tell they are exceeding this level?    3. After you identify they are drinking too much, then what do you typically do?    4. How do you decide what to do?    5. Who else do you think needs to be involved (e.g., other clinicians, family members, etc.)? 4. **Tell me about your experiences evaluating patients with unhealthy alcohol use for Hepatitis C treatment.**    1. What about for patients with other liver conditions?    2. What resources and/or treatments do you offer?    3. Do you have any concerns? 5. **If your clinic were to do more to address unhealthy alcohol use at the point of treatment for HCV or other liver conditions, what would be needed?** 6. **From the perspective of clinic flow, what would be the benefits and/or drawbacks?** 7. **Do you think your clinic leadership would be supportive of doing more to address unhealthy alcohol use?**   ***Interviewer:*** *Thank you. This is the last section. For the next few minutes, I’d like to get your feedback on some ideas we have about how we could help support providing care for unhealthy alcohol use in the Hepatology clinic.*  *(Point interviewee to the handout, which will replicate a-e below without prompts to solicit feedback)*   1. **We would like to hear from you regarding whether these elements would be helpful and also whether they would be feasible to implement? Why or why not?**    1. Having a point person from our team help form a local team with a clinical champion. The local clinical team would be trained in evidence-based care for unhealthy alcohol and other substance use, and we would help you redesign clinical workflow to facilitate this care in the clinic.       1. Our vision is that this will involve participating in 3 successive “design meetings” of decreasing lengths (~3 hours, ~2 hours, and ~1.5 hours), but we wonder what might work best for you.  - Would this be useful? - Do you think this would be feasible?   1. After initial design meetings, the point person from our team would facilitate monthly teleconferences with the local clinical team to troubleshoot ongoing issues. - Would this be useful? - Do you think this would be feasible?   1. We would provide the local clinical team, and the clinic as a whole, patient-centered educational materials, including both hand-outs and a YouTube video on this reframe. These materials can be used with patients, and as training for staff. - Would this be useful? - Do you think this would be feasible?   1. We may be able to develop informatics tools to prompt and document evidence-based care. These could include:      1. An “order set” that will walk a clinician through several different ways to approach treatment for unhealthy alcohol use, and if desired will facilitate provision of a prescription for AUD pharmacotherapy      2. A “consult menu” that will easily enable a referral to specialty addictions treatment - Would this be useful? - Do you think this would be feasible?   1. We hope to provide real-time data on a weekly or monthly basis to clinics to help monitor performance on provision of care for unhealthy alcohol use. - Would this be useful? - Do you think this would be feasible?  1. **Is there anything else you’d like to tell us?** |
| --- |
